# Experimental Realization of an Extreme-Parameter Omnidirectional Cloak

**DOI:** 10.34133/2019/8282641

**Published:** 2019-08-18

**Authors:** Bin Zheng, Yihao Yang, Zheping Shao, Qinghui Yan, Nian-Hai Shen, Lian Shen, Huaping Wang, Erping Li, Costas M. Soukoulis, Hongsheng Chen

**Affiliations:** ^1^Key Lab. of Advanced Micro/Nano Electronic Devices & Smart Systems of Zhejiang, College of Information Science and Electronic Engineering, Zhejiang University, Hangzhou 310027, China; ^2^State Key Laboratory of Modern Optical Instrumentation and The Electromagnetics Academy at Zhejiang University, Zhejiang University, Hangzhou 310027, China; ^3^Department of Physics and Astronomy and Ames Laboratory-U.S. DOE Iowa State University, Ames, IA 50011, USA; ^4^Institute of Marine Electronics Engineering, Zhejiang University, Hangzhou 310058, China; ^5^Institute of Electronic Structure and Laser, FORTH, 71110 Heraklion, Crete, Greece

## Abstract

An ideal transformation-based omnidirectional cloak always relies on metamaterials with extreme parameters, which were previously thought to be too difficult to realize. For such a reason, in previous experimental proposals of invisibility cloaks, the extreme parameters requirements are usually abandoned, leading to inherent scattering. Here, we report on the first experimental demonstration of an omnidirectional cloak that satisfies the extreme parameters requirement, which can hide objects in a homogenous background. Instead of using resonant metamaterials that usually involve unavoidable absorptive loss, the extreme parameters are achieved using a nonresonant metamaterial comprising arrays of subwavelength metallic channels manufactured with 3D metal printing technology. A high level transmission of electromagnetic wave propagating through the present omnidirectional cloak, as well as significant reduction of scattering field, is demonstrated both numerically and experimentally. Our work may also inspire experimental realizations of the other full-parameter omnidirectional optical devices such as concentrator, rotators, and optical illusion apparatuses.

## 1. Introduction

Various design concepts of invisibility cloaks have been recently realized thanks to the pioneering works in metamaterials and transformation optics. Designs of invisibility cloaks based on other methods have also been reported [1–3]. From the viewpoint of transformation optics, a concealed region is mapped from a point in virtual space. Despite the elegance of the proposed general transformation optics cloaking theory [4, 5], it is tremendously difficult to implement the required extreme parameters, and this is why previous implementations have relied on approximations [6–8]. These approximations degrade the performance of the cloak due to considerable reflections. Another way to eliminate the extreme parameters is to transform the hidden object into a line instead of a point, the idea behind carpet cloaks [9–16] and unidirectional cloaks [17–19]. However, the carpet cloaks should work on a conducting ground plane and the unidirectional cloaks suffer from narrow viewing angles. In short, an omnidirectional cloak with perfect performance requires extreme parameters but, still, remains elusive.

All of these difficulties stem from the need to create a hidden region inside the cloak that does not exist before the transformation. The hidden region has a finite dimension, but it is mapped from a point with an infinitesimal size. The light rays must therefore propagate around the inner boundary of the cloak with an infinite speed, and the constitutive parameters need to be of extreme values,* e.g.*, *ε*_*ρ*_(*μ*_*ρ*_) = 0 and *ε*_*θ*_(*μ*_*θ*_) = *∞* (the so-called singularity) for transverse magnetic TM (transverse electric TE) waves at the inner boundary of the cylindrical cloaks [20]. These extreme parameters cannot be avoided for an ideal omnidirectional cloak with perfect performance, independently of the choice of coordinate transformation equations. The designs of metamaterials with permeability (TE wave case) or permittivity (TM wave case) ranging from zero to infinity are very challenging in general. Resonant metamaterials, such as SRRs [21], usually suffer from material absorptions that prevent the permeability from reaching an infinite value. Therefore, the extreme parameters requirement is one of the main challenge that hinders the realization of omnidirectional cloaks with perfect performance.

Here, we successfully achieved the first practical realization of an omnidirectional cloak with extreme parameters that can hide an object in a homogenous isotropic background. We subtly design a metamaterial to fulfill the extreme parameters requirement by taking advantage of the nature of the conductive metals at low frequencies. Instead of using resonant metamaterials, we realize all the constitutive parameters with non-resonant metamaterials that display lower material absorptions. As a demonstration, we fabricate (with the aid of three-dimensional (3D) metal printing technology) and experimentally characterize a metamaterial cloak sample.

## 2. Results and Discussion

**Figure 1:**
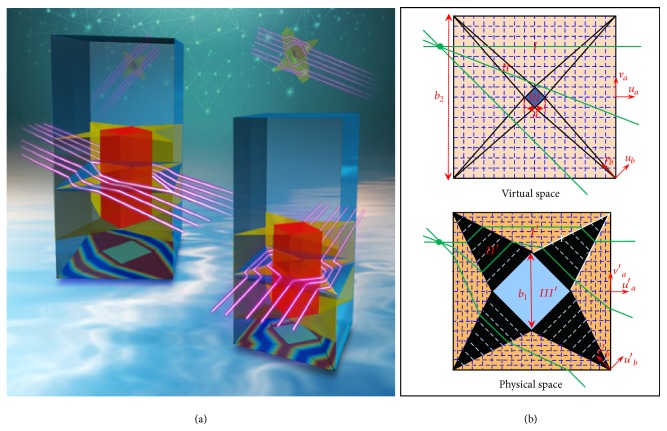
(a) Schematic view of the proposed omnidirectional cloak with extreme parameters. (b) Designing an omnidirectional cloak based on transformation optics. The space between the big square and the small one in virtual space is transformed into corresponding regions in physical space. These regions are divided into eight triangular segments that can be grouped into two groups: Region I (I′) and Region II (II′). The green lines represent trajectories of rays. The blue square is the hidden area.

Figure 1(a) shows the schematic view of the proposed omnidirectional cloak with extreme parameters. In a cylindrical cloak, a point in the virtual space is transformed into a circle in the physical space and different circles have different tension or compression ratios that lead to constitutive parameters of the cloak spatially distributed along the radial direction. In the present work, instead of using an inhomogeneous transformation in a cylindrical cloak, we divided the cloak shell into eight regions and used a homogeneous transformation [22] to design a square cloak, as shown in Figure 1(b). The space between a square of side *b*_2_ and a smaller square of side *a* in the virtual space is transformed into the space between a square of side *b*_2_ and a smaller square of side *b*_1_ in the physical space. Here, we set *a* = 0, i.e., the square in the virtual space with zero cross section, and *b*_2_ = 2*b*_1_. For the TM wave case and embedding the cloak in a homogenous background with *ε*_*bg*_ = 1 and *μ*_*bg*_ = 1, by using transformation optics (see Supplementary Information ([Supplementary-material supplementary-material-1])), we obtain the constitutive parameters (for simplification, we use superscript* u*,* v*,* w* instead of* u*′,* v*′,* w*′ in physical space): *ε*_*a*_^*u*^ = 0.5, *ε*_*a*_^*v*^ = 2, *μ*_*a*_^*w*^ = 2 in Region I′, and *ε*_*b*_^*u*^ = *∞*, *ε*_*b*_^*v*^ = 0, *μ*_*b*_^*w*^ = 0 in Region II′; an object in Region III′ becomes completely invisible from all of the view angles. It is worth noting that region II′ in physical space is now an optical nihility medium [23–25] with extreme anisotropic parameters, which is mapped from a volumeless line in the virtual space. The region I′ is with regular anisotropic medium while the region III′ is the hidden region for arbitrary objects. In this cloak, while zero and infinite values are still present, the large values close to the singularity vanish. This will facilitate the experimental implementation but without losing any performance. Note that the proposed cloak preserves both the phase and amplitude of the light, which is different from the cloak designed with geometrical optics [26].

The key point for realizing this cloak lies on how to design an appropriate metamaterial [27, 28] to provide the constitutive parameters of Region II′, that include both zero and infinite values. Previous work has shown that one dimensional metallic silt array can find equivalency to this nihility medium right at the Fabry-Pérot (FP) resonant frequency [25, 29]. However, this FP resonant frequency will be related with the propagation length in the* u*′ directions, making it not suitable for the realization of the Region II′. In order to do this, we should first understand the physical nature of this medium. It is obvious that in Region II′, electromagnetic (EM) waves cannot propagate along the* v*′ direction, because *ε*_*b*_^*u*^ = *∞* and the material behaves like a perfect electric conductor (PEC). However, since *ε*_*b*_^*v*^ = 0, *μ*_*b*_^*w*^ = 0 EM waves can propagate along the* u*′ direction, with infinite phase velocity or zero phase delay. The Region II′ medium behaves like a sampler that samples the phase and amplitude of EM waves along one boundary (*e.g. *the white line marked 1 in Figure 1(c)) and then transfers this information, including phase and amplitude, to the other boundary (*e.g. *the white line marked 2 in Figure 1(c)), or vice versa. In fact, the whole square of side *b*_2_ in the virtual space is transformed into I′-type regions in the physical space. The II′-type regions are nihility spaces that play the role of connecting each I′-type region.

**Figure 2:**
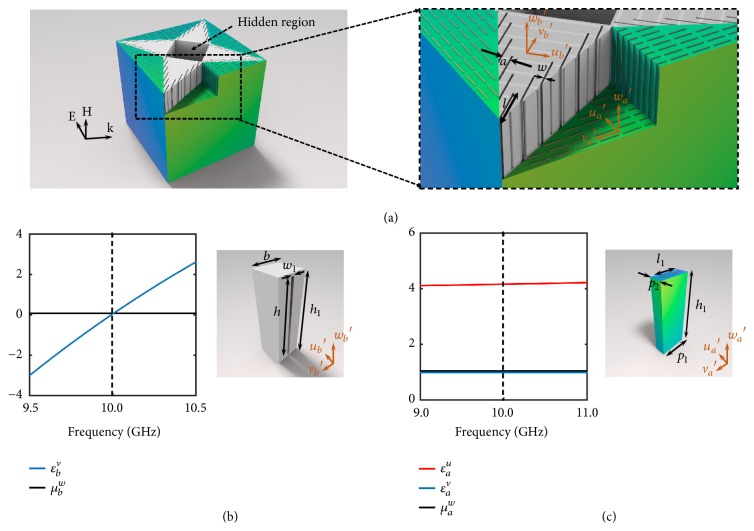
*Unit cell designs of the omnidirectional metamaterial cloak*. (a) Designed metamaterial cloak. (b) Unit cell and the corresponding effective constitutive parameters of the extreme metamaterial. Here, *h*=15.0 mm, *h*_1_=16.0 mm, *b*=5.0 mm, and the width of the slits is *w*_1_=0.25 mm. (c) Unit cell and the corresponding effective constitutive parameters of the non-extreme metamaterial. Here, *p*_1_= 6.0 mm, *p*_2_=2.35 mm, and *l*_1_=5.0 mm.

With this profound physical understanding in mind, we successfully design a metamaterial composed of metallic channel and slits arrays that exhibit the same behavior of the II′-type medium (see Figure 2(b)). In this metamaterial, the effective relative permittivity is *ε*_*b*_^*v*^ = *n*_0_^2^ − *c*^2^/(4*f*^2^*h*^2^), where *n*_0_ is the refraction index of air, *c* is the speed of light in the free space, *f* is the frequency, and *h* is the height of the metal channel [27]. Therefore, at the cut-off frequency of the metallic channels, *ε*_*b*_^*v*^ = 0. If the width of the channels is sufficiently narrow, the EM waves can tunnel through the waveguide with almost total transmission and zero phase delay, behaving exactly like the epsilon-mu-near-zero medium [30–32]. We apply a well-established retrieval process [33] to obtain the effective constitutive parameters, showing that around 10.0 GHz, both *ε*_*b*_^*v*^ and *μ*_*b*_^*w*^ are exactly zero. This metallic channel array metamaterial automatically fulfills the requirement of *ε*_*b*_^*u*^ = *∞*, because EM waves are prevented to propagate along the* v*′ direction by the PEC walls. Therefore, we achieve the singular parameter by simply making the most of the nature of the conductive metals at low frequency.

In order to destroy the spoof surface plasmon polaritons induced by the metallic channel arrays [34–36], we add some splits along the* v*′ direction on the surface of Region II′ (see the inset of Figure 2(a)). From a metamaterial perspective, this slit array has a macroscopic effective EM response, namely, *ε*_*b*_^*u*^ = *a*/*w*, *μ*_*b*_^*w*^ = *w*/*a*, where *a* and *w* are respectively the period and width of the slits. When *w* ≪ *a* ≪ *λ*_0_, then *ε*_*b*_^*u*^ → *∞* and *μ*_*b*_^*w*^ → 0; therefore, this structure fulfills exactly the parameter requirements in Region II′. In this experimental realization, we choose *a*=2.35 mm, *w*=0.5 mm, and *l*=10.0 mm. These self-supporting metallic channel arrays is fabricated by a standard commercial 3D printing using an aluminum alloy.

The constitutive parameters of medium for Region I′ is finite; i.e., *ε*_*a*_^*u*^ = 0.5, *ε*_*a*_^*v*^ = 2, and *μ*_*a*_^*w*^ = 2. For easy of realization, we use an ultrathin cut-wire metamaterial to construct this medium, as shown in Figure 2(c). The cut wires are print on an ultrathin printed circuit board (PCB) (0.1 mm Teflon woven glass fabric copper-clad laminate with a permittivity of 2.5 and tg*δ* < 0.001 at 10 GHz). Then we attach them on polymer foam sheets (1.06 mm Rohacell 71HF with a permittivity of 1.1 and tg*δ* < 0.0016 at 10.0 GHz) and stack them layer-by-layer constructing a bulky metamaterial. The retrieved effective constitutive parameters at 10.0 GHz, *ε*_*a*_^*u*^ = 1, *ε*_*a*_^*v*^ = 4, *μ*_*a*_^*w*^ = 1, fulfilling the parameters requirement for Region I′. Physically, at frequencies much lower than the resonance frequency, the cut-wire metamaterial provides a paraelectric response along the cut wires (*ε*_*a*_^*v*^ > 1) and the interaction with the electric field perpendicular to the cut wires is very weak (*ε*_*a*_^*u*^ = 1). In addition, as the cut wires create only an electric response, the effective permeability remains almost unity (*μ*_*a*_^*w*^ = 1). We can obtain the desired EM responses by tuning the geometries of the cut wires,* i.e.*, *p*_1_, *p*_2_, and *l*_1_.

It is interesting to notice that both of the conjoint metamaterials are non-resonant, whose benefits are threefold. First, as the EM responses of the metamaterials are far below resonance, the losses are inherently low. Second, the sizes of the unit cells can be as small as needed,* i.e.*, we can shrink the sizes of unit cells proportionally in the *uv*-plane and the constitutive parameters at 10.0 GHz won't change. Thus, if the unit cells are small enough, at the intersection of different regions all of the boundaries can match very well with negligible voids in the implementation. Last but not least, while resonant metamaterials are generally sensitive to small fabrication imperfections that may dramatically change their EM properties, nonresonant metamaterials [37] are reliably robust to parametric uncertainties.

**Figure 3:**
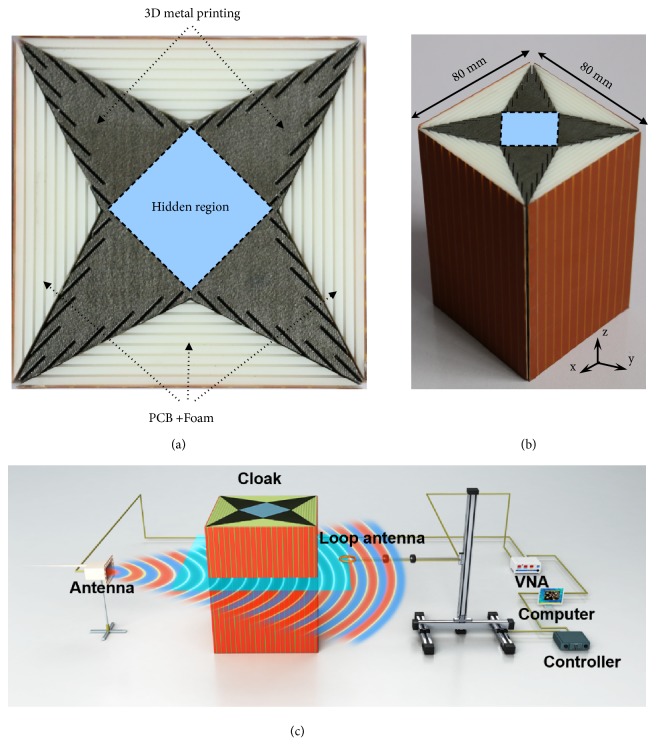
*Photographs of the fabricated cloak and scheme of the experiment setup*. (a) Top view of the metamaterial cloak. The extreme metamaterial is realized with the standard commercial 3D metal printing technology and the non-extreme one is implemented with the PCB-Foam layered structures. (b) Perspective view of the metamaterial cloak with 8 unit cells in the *z* direction. (c) Scheme of the experiment setup. An open X-band rectangular waveguide with z-polarized magnetic field, located at a distance of 50 mm away from the cloak, is used as a source. A loop antenna with a radius of 4 mm is used as the receiving antenna. Both transmitting and receiving antennas are connected to a VNA to obtain the amplitude and phase of the measured field. The measuring loop antenna is attached to a mechanical arm of a 3D measurement platform and can be controlled to move in the xy planes to point-to-point probe the magnetic field. The scan region (the light-blue rectangle) is 300 mm by 236 mm with a resolution of 4 mm.

The fabricated cloak sample (shown in Figure 3) is 80 mm by 80 mm, with eight unit cells (the height of each unit cell is 17 mm) in the* z*-direction. With a preliminary simulation (see Supplementary Information), we find that the cloak can be very robust with only a few unit cells in the* z-*direction. On the one hand, as the metallic channel metamaterial confines the energy inside, the couplings between neighboring channels in the* z-*direction are quite weak. On the other hand, the cut-wire metamaterial is also not sensitive to the variances in the* z*-direction.

The proposed cloak works for a background with *ε*_*bg*_ = 2 and *μ*_*bg*_ = 0.5,* i.e.*, a refractive index of* n*=1, the same as that in air. In the measurement, for simplicity, we replace the background with air, causing some minor reflections at the outer boundary of the omnidirectional cloak without altering the extreme parameters requirement in the omnidirectional cloak. These reflections, due to the mismatch of the impedance at the outer boundary, can be technically solved by adding an isotropic gradient metamaterial layer around the cloak, whose refraction index stays unity and impedance changes slowly from that of air to that of the metamaterial of the cloak (a detailed design of this gradually changing metamaterial layer is included in the Supplementary Information). In fact, this method has been applied in some other cloaking designs, such as the full-parameter unidirectional cloak, in which a taper structure with gradually changing impedance is used to couple the EM waves from the air to the cloak region [19].

We set up an experimental system to measure the *H*_*z*_-field distributions around the cloak in a fully anechoic chamber, as shown in Figure 3(c). An open X-band rectangular waveguide with z-polarized magnetic field, located at a distance of 50 mm away from the cloak, is used as a source. A loop antenna with a radius of 4 mm is used as the receiving antenna. Both transmitting and receiving antennas are connected to a vector network analyzer (VNA) to obtain the amplitude and phase of the measured field. The measuring loop antenna is attached to a mechanical arm of a 3D measurement platform and can be controlled to move in the xy planes to point-to-point probe the magnetic field. The scan region (the light-blue rectangle) is 300 mm by 236 mm with a resolution of 4 mm.

**Figure 4:**
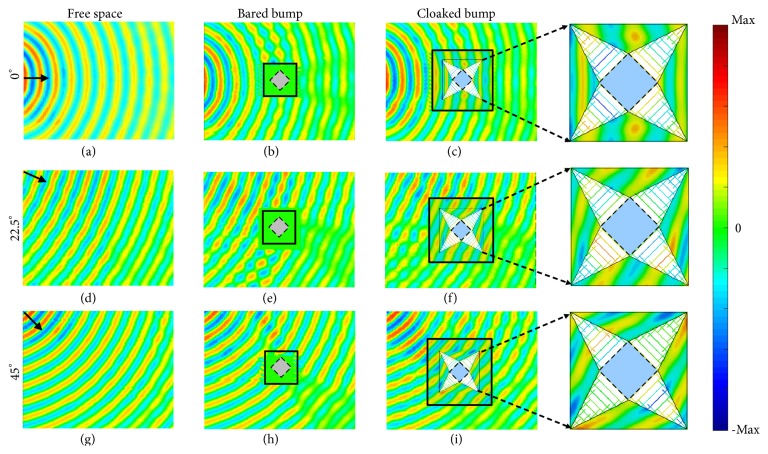
*Measured H*_*z*_ *magnetic field distributions near the metamaterial cloak at the optimum cloaking frequency of 9.8 GHz*. Field distributions for free space, bared aluminum cylinder and cloaked aluminum cylinder, respectively, when EM waves are incident with an angle of 0° [(a)–(c)], 22.5° [(d)–(f)], and 45° [(g)–(i)], respectively. The black-line squares and the gray areas in (b), (e), and (h) represent the unmeasured region and the aluminum cylinder, respectively. The black arrows in (a), (d), and (g) indicate the incidence of the point source of EM waves. The bold black line squares and the blue squares in (c), (f), and (i) represent the unmeasured and hidden regions, respectively. The unmeasured areas are filled with simulated *H*_*z*_-field distributions. Insets: the enlarged image of *H*_*z*_*-*field distributions in the cloak.

In the experiments, we measure three cases, which are with an incidence angle of 0° (Figures 4(a)–4(c)), 22.5° (Figures 4(d)–4(f)), and 45° (Figures 4(g)–4(i)), respectively. The optimum cloaking frequency of the metamaterial cloak slightly shifts from 10.0 GHz to 9.8 GHz in the implementation. From the measured results one can see that placing a bared PEC bump in the free space causes strong reflections (backward scattering) and forms a shadow behind the bump (forward scattering) (Figures 4(b), 4(e), and 4(h)). The forward and backward scatterings are strongly suppressed by covering the bump with a cloak ((Figures 4(c), 4(f), and 4(i)), where both the phase and amplitude of the EM wave are well reconstructed. As we get rid of the background, some reflections reasonably occur, resulting from the impedance mismatching. Note that the bold solid squares in Figures 4(c), 4(f), and 4(i) represent the unmeasured regions. To get complete information in these regions, we fill the unmeasured regions with the simulated magnetic field distributions. The simulated and experimental results are in excellent agreement. From the results, we can exactly see that the EM waves are first separated into two beams, that pass through Region I′ with a compressed wavelength and tunnel through the metallic rectangular waveguide with zero-phase delay (see the insets), then rejoin again with the same phase and amplitude, as if they had passed directly through the air. As the effective singular parameter of *ε*_*b*_^*v*^ = 0 occurs at cut-off frequency, the cloak still works at single frequency. Nevertheless, it will be the first step to achieve extreme parameter cloak with omnidirectional cloaking performance robustly because both Region I′ and II′ are realized with non-resonant homogeneous metamaterials with pretty simple structures and the absorptions of these metamaterials are weak.

## 3. Conclusions and Outlook

In summary, we design, fabricate and experimentally characterize the first extreme-parameter omnidirectional cloak with non-resonant metamaterials. Both of the numerical and experimental results clearly show the excellent performance of omnidirectional invisibility of the present cloak, verifying the rightness of our design. The present work provides an important guidance toward the realization of complex functionalities with metamaterials, and will motivate various transformation optics based devices for practical applications, such as concentrators [38, 39], rotators [40], and optical illusion [41] apparatuses.

## Supplementary Materials

Supplementary Materials Figure S1.* Full-wave simulation of the full-parameter omnidirectional cloak with practical structures.* (a)-(d) Magnetic field distributions near the present cloak when a point source emits EM wave along the side (a), the diagonal (b), and a nonsymmetry plane (c) of the cloak, and the side of a 10-wavelength-size cloak (d), respectively. The blue squares represent the hidden regions. (e)-(h) *H*_*z*_-field patterns when both the PEC object and the cloak are removed from the homogenous background. (i)-(l) *H*_*z*_-field patterns when only the PEC object is present without the cloak. The gray squares represent the PEC objects. (m)-(o) Differential RCS of the present cloak (the red line) and bared PEC objects (the blue line) at 10.0 GHz when the EM wave is incident at an angle of 0°, 45°, and 22.5°, respectively. The reduced total RCS of the three cases are 0.0829, 0.0717, and 0.0743, respectively. (p) Reduced total RCS as a function of frequency at the incidence angle of 45°. Figure S2. (a)-(b) A mismatched-impedance material (*ε*_*b*_ = 2, *μ*_*b*_ = 0.5) covered with the GML. (c)-(d) A bared mismatched-impedance material. (e)-(f) A mismatched-impedance material (*ε*_*b*_ = 2,*μ*_*b*_ = 0.5) covered with the discretized GML. (g)-(h) The omnidirectional cloak covered with the GML. Figure S3. (a)-(b) A mismatched-impedance material (*ε*_*b*_ = 2,*μ*_*b*_ = 0.5) covered with the discrete GML composed of practical structures. (c)-(d) Without the discrete GML. (e) Metamaterial unit cell. It is a rectangular metallic waveguide loaded with a dielectric material (*ε*_*l*_ = 2.5). The period of the unit cell is *p*, and between each unit cell is air. (f) Geometries (the unit is mm), targeted relative permittivity (*ε*) and permeability (*μ*), and effective relative permittivity (*ε*′) and permeability (*μ*′) of each unit cell. Here, *h*_1_=16 mm, *a*=3 mm, and *p*=4 mm are fixed for all unit cells.
