# Supplementary material for: Experimental Realization of an Extreme-Parameter Omnidirectional Cloak
Source: Research (Wash D C). 2019 Aug 18;2019:8282641. doi: 10.34133/2019/8282641 (PMC6750086; doi:10.34133/2019/8282641)
Supplement: Supplementary Materials — Figure S1. Full-wave simulation of the full-parameter omnidirectional cloak with practical structures. (a)-(d) Magnetic field distributions near the present cloak when a point source emits EM wave along the side (a), the diagonal (b), and a nonsymmetry plane (c) of the cloak, and the side of a 10-wavelength-size cloak (d), respectively. The blue squares represent the hidden regions. (e)-(h) Hz-field patterns when both the PEC object and the cloak are removed from the homogenous background. (i)-(l) Hz-field patterns when only the PEC object is present without the cloak. The gray squares represent the PEC objects. (m)-(o) Differential RCS of the present cloak (the red line) and bared PEC objects (the blue line) at 10.0 GHz when the EM wave is incident at an angle of 0°, 45°, and 22.5°, respectively. The reduced total RCS of the three cases are 0.0829, 0.0717, and 0.0743, respectively. (p) Reduced total RCS as a function of frequency at the incidence angle of 45°. Figure S2. (a)-(b) A mismatched-impedance material (εb = 2, μb = 0.5) covered with the GML. (c)-(d) A bared mismatched-impedance material. (e)-(f) A mismatched-impedance material (εb = 2,μb = 0.5) covered with the discretized GML. (g)-(h) The omnidirectional cloak covered with the GML. Figure S3. (a)-(b) A mismatched-impedance material (εb = 2,μb = 0.5) covered with the discrete GML composed of practical structures. (c)-(d) Without the discrete GML. (e) Metamaterial unit cell. It is a rectangular metallic waveguide loaded with a dielectric material (εl = 2.5). The period of the unit cell is p, and between each unit cell is air. (f) Geometries (the unit is mm), targeted relative permittivity (ε) and permeability (μ), and effective relative permittivity (ε′) and permeability (μ′) of each unit cell. Here, h1=16 mm, a=3 mm, and p=4 mm are fixed for all unit cells. [file 8282641.f1.zip › 8282641_SupplDesc..docx]

**Figure S1.** **Full-wave simulation of the full-parameter omnidirectional cloak with practical structures.** (a)-(d) Magnetic field distributions near the present cloak when a point source emits EM wave along the side (a), the diagonal (b), and a non-symmetry plane (c) of the cloak, and the side of a 10-wavelength-size cloak (d), respectively. The blue squares represent the hidden regions. (e)-(h) *Hz*-field patterns when both the PEC object and the cloak are removed from the homogenous background.(i)-(l) *Hz*-field patterns when only the PEC object is present without the cloak. The gray squares represent the PEC objects. (m)-(o) Differential RCS of the present cloak (the red line) and bared PEC objects (the blue line) at 10.0 GHz when the EM wave is incident at an angle of 0°, 45°, and 22.5°, respectively. The reduced total RCS of the three cases are 0.0829, 0.0717, and 0.0743, respectively. (p) Reduced total RCS as a function of frequency at the incidence angle of 45°.

**Figure S2.** (a)-(b) A mismatched-impedance material (,) covered with the GML. (c)-(d) A bared mismatched-impedance material. (e)-(f) A mismatched-impedance material (,) covered with the discretized GML. (g)-(h) The omnidirectional cloak covered with the GML.

**Figure S3.** (a)-(b) A mismatched-impedance material (,) covered with the discrete GML composed of practical structures. (c)-(d) Without the discrete GML. (e) Metamaterial unit cell. It is a rectangular metallic waveguide loaded with a dielectric material (). The period of the unit cell is, and between each unit cell is air. (f) Geometries (the unit is mm), targeted relative permittivity () and permeability (), and effective relative permittivity () and permeability () of each unit cell. Here, =16 mm, =3 mm, and =4 mm are fixed for all unit cells.
