# Supplementary material for: Experimental Realization of an Extreme-Parameter Omnidirectional Cloak
Source: Research (Wash D C). 2019 Aug 18;2019:8282641. doi: 10.34133/2019/8282641 (PMC6750086; doi:10.34133/2019/8282641)
Supplement: Supplementary Materials — Figure S1. Full-wave simulation of the full-parameter omnidirectional cloak with practical structures. (a)-(d) Magnetic field distributions near the present cloak when a point source emits EM wave along the side (a), the diagonal (b), and a nonsymmetry plane (c) of the cloak, and the side of a 10-wavelength-size cloak (d), respectively. The blue squares represent the hidden regions. (e)-(h) Hz-field patterns when both the PEC object and the cloak are removed from the homogenous background. (i)-(l) Hz-field patterns when only the PEC object is present without the cloak. The gray squares represent the PEC objects. (m)-(o) Differential RCS of the present cloak (the red line) and bared PEC objects (the blue line) at 10.0 GHz when the EM wave is incident at an angle of 0°, 45°, and 22.5°, respectively. The reduced total RCS of the three cases are 0.0829, 0.0717, and 0.0743, respectively. (p) Reduced total RCS as a function of frequency at the incidence angle of 45°. Figure S2. (a)-(b) A mismatched-impedance material (εb = 2, μb = 0.5) covered with the GML. (c)-(d) A bared mismatched-impedance material. (e)-(f) A mismatched-impedance material (εb = 2,μb = 0.5) covered with the discretized GML. (g)-(h) The omnidirectional cloak covered with the GML. Figure S3. (a)-(b) A mismatched-impedance material (εb = 2,μb = 0.5) covered with the discrete GML composed of practical structures. (c)-(d) Without the discrete GML. (e) Metamaterial unit cell. It is a rectangular metallic waveguide loaded with a dielectric material (εl = 2.5). The period of the unit cell is p, and between each unit cell is air. (f) Geometries (the unit is mm), targeted relative permittivity (ε) and permeability (μ), and effective relative permittivity (ε′) and permeability (μ′) of each unit cell. Here, h1=16 mm, a=3 mm, and p=4 mm are fixed for all unit cells. [file 8282641.f1.zip › Supplementary Materials.docx]

**Supplementary materials for**

**Experimental Realization of an Extreme-Parameter Omnidirectional Cloak**

1. **Omnidirectional cloak based on a linear homogeneous transformation**

We divide a square in the virtual space into several regions and transform them to their corresponding regions in the physical space as shown in Fig. 1 (a) and (b), *i.e.*, Region I (with local coordinate systems ) to Region I’ (with local coordinate systems ), Region II (with local coordinate systems ) to Region II’ (with local coordinate systems). The coordinate transformation in each region is as following:

, for Region I’, and

, for Region II’,

where , , and are the compression or extension ratio of the corresponding regions. For a homogenous virtual space with a permittivity of and a permeability of , and for a TM polarization, we can get the constitutive parameters in each region in the physical space:

, for Region I’, and

, for Region II’.

Setting , , and assuming a virtual space with , the constitutive parameters in each region become (for simplification, we use superscript *u*, *v*, *w* instead of *u’*, *v’*, *w’* in physical space)

, for Region I’, and

, for Region II’.

1. **Full-wave simulation of the omnidirectional cloak**

We perform a series of full-wave simulations in the CST to verify the cloaking performance guaranteed by the transformation optics methods. The cloak with the real structure of the extreme metamaterial is used in the simulation. A point source at different locations around the cloaked PEC object is used to illuminate the cloak sample (Fig. S1 (a)-(c)), the homogenous background (Fig. S1 (e)-(g)), and the uncloaked PEC object (Fig. S1(i)-(k)), respectively. As a point source contains many plane wave components with different direction, it is beneficial to the demonstration of the omnidirectionality of the cloak. From the *Hz*-field patterns, one can see that the present cloak can dramatically reduce the scattering induced by the PEC object omnidirectionally. We also simulate a 10-wavelength-scale omnidirectional cloak (Fig. S1(d), (h) and (l)), the results clearly demonstrate the excellent cloaking performance of the cloak, which is difficultly achieved with the other methods. We calculate the radar cross sections (RCSs) of the cloaked object and the bared one at different incident angles (Fig. S1(m), (n), and (o)), respectively. One can see that the cloak strongly suppresses the scattering of the object from all of the view angles. Besides, the reduced total RCSs (the total RCSs of the cloaked object divided by that of the bared object) reach to less than 10% without regard to the incident angles. Finally, we plot the reduced total RCSs as a function of the frequency at the incident angle of 45° and the total RCS dramatically decreases at 10.0 GHz.


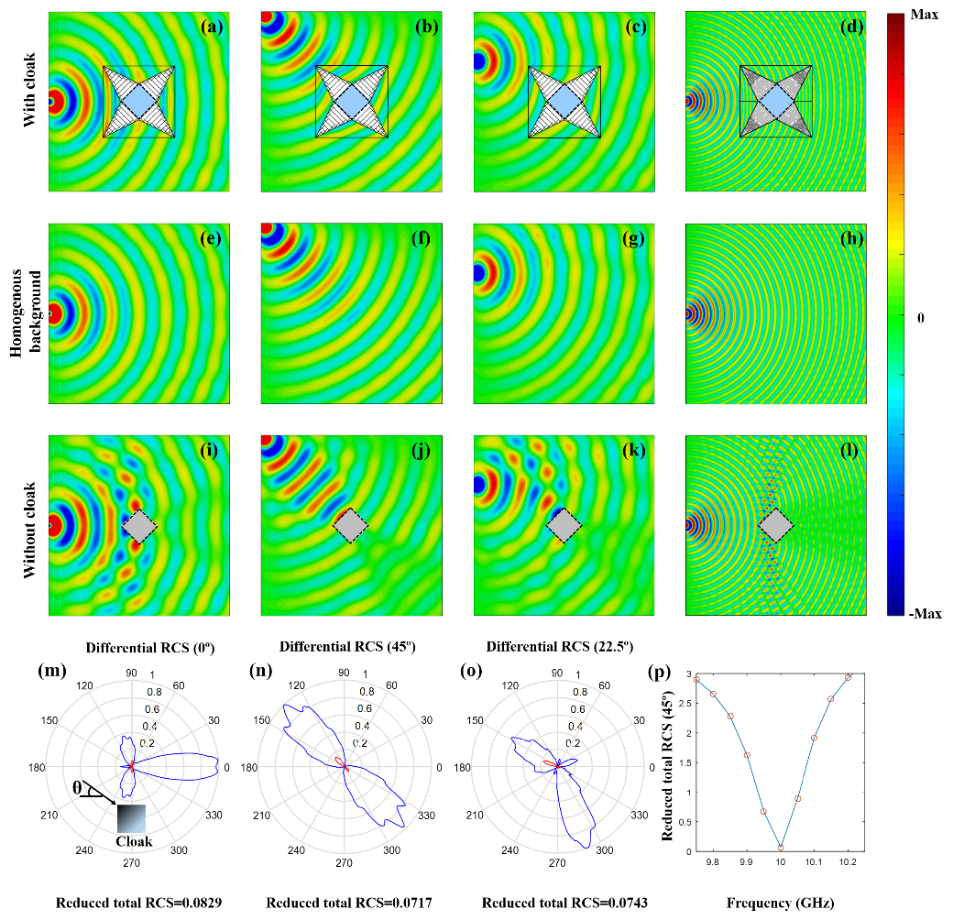


**Figure S1.** **Full-wave simulation of the full-parameter omnidirectional cloak with practical structures.** (a)-(d) Magnetic field distributions near the present cloak when a point source emits EM wave along the side (a), the diagonal (b), and a non-symmetry plane (c) of the cloak, and the side of a 10-wavelength-size cloak (d), respectively. The blue squares represent the hidden regions. (e)-(h) *Hz*-field patterns when both the PEC object and the cloak are removed from the homogenous background.(i)-(l) *Hz*-field patterns when only the PEC object is present without the cloak. The gray squares represent the PEC objects. (m)-(o) Differential RCS of the present cloak (the red line) and bared PEC objects (the blue line) at 10.0 GHz when the EM wave is incident at an angle of 0°, 45°, and 22.5°, respectively. The reduced total RCS of the three cases are 0.0829, 0.0717, and 0.0743, respectively. (p) Reduced total RCS as a function of frequency at the incidence angle of 45°.

1. **Gradient metamaterial layer**

In the gradient metamaterial layer (GML), the refraction index keeps the same, while the impedance gradually changes from that of background to that of the central region. As shown in Fig. S2(a)-(b), with the GML, the scattering caused by the impedance matching can be dramatically reduced, comparing with the results without the GML (Fig. S2(c)-(d)). Here, the size of the central square (, ) is 80 mm by 80 mm; the thickness of the GML is 32 mm and the relative impedance linearly changes from 0.5 to 1; the operational frequency is 10 GHz. By wrapping our omnidirectional cloak with the GML, the slight reflection caused by the impedance mismatched between the free space and the cloak can be further reduced and the cloak works with almost perfect performance in the free space (Fig. S2(g)-(h)).

To show its feasibility in implementation, we first discretize it into four layers and they still work very well (Fig. S2(e)-(f)); then we design a metamaterial unit cell (Fig. S3(e)) to realize the required constitutive parameters. The unit cell is a metallic rectangular waveguide structure loaded with a dielectric material (). The period of the unit cell is, and between each unit cell is air.Properly designing the geometries, we obtain the desired electromagnetic responses. The geometries, targeted relative permittivity () and permeability (), and effective relative permittivity () and permeability () of each unit cell are shown in Fig. S3(f). Covered the mismatched-impedance material (, ) with a practical GML, the scatterings are further reduced as wanted (Fig. S3(a)-(b)).


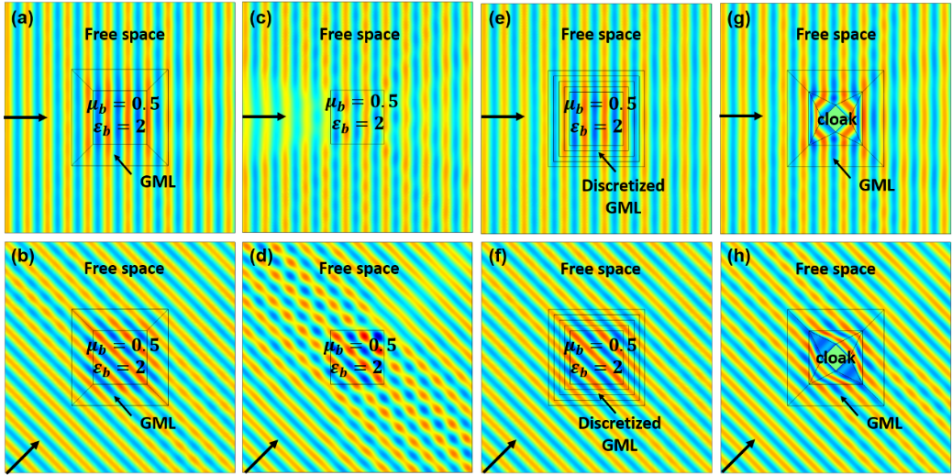


**Figure S2.** (a)-(b) A mismatched-impedance material (,) covered with the GML. (c)-(d) A bared mismatched-impedance material. (e)-(f) A mismatched-impedance material (,) covered with the discretized GML. (g)-(h) The omnidirectional cloak covered with the GML.


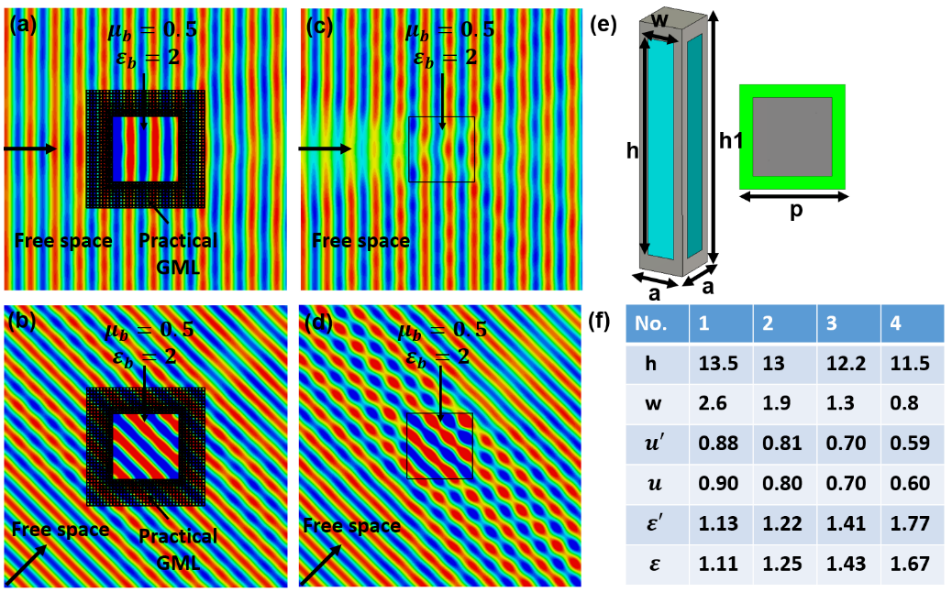


**Figure S3.** (a)-(b) A mismatched-impedance material (,) covered with the discrete GML composed of practical structures. (c)-(d) Without the discrete GML. (e) Metamaterial unit cell. It is a rectangular metallic waveguide loaded with a dielectric material (). The period of the unit cell is, and between each unit cell is air. (f) Geometries (the unit is mm), targeted relative permittivity () and permeability (), and effective relative permittivity () and permeability () of each unit cell. Here, =16 mm, =3 mm, and =4 mm are fixed for all unit cells.
